# Phosphatidylcholine (PCL) fortified nano-phytopharmaceuticals for improvement of therapeutic efficacy

**DOI:** 10.17179/excli2023-6345

**Published:** 2023-08-18

**Authors:** Devesh U. Kapoor, Mansi Gaur, Akshay Parihar, Bhupendra G. Prajapati, Sudarshan Singh, Ravish J. Patel

**Affiliations:** 1 Dr. Dayaram Patel Pharmacy College, Bardoli-394601 Gujarat, India; 2 Senior Process Associate, Medical Scribe, Integrity Healthcare Solutions, Ahmedabad-380054, Gujarat, India; 3 Faculty of Pharmaceutical Sciences, The ICFAI University, Baddi-174103, Himachal Pradesh, India; 4 Shree S.K. Patel College of Pharmaceutical Education and Research, Ganpat University, Mehsana-384012, Gujarat, India; 5 Office of Research Administration, Chiang Mai University, Chiang Mai 50200, Thailand; 6 Department of Pharmaceutical Sciences, Faculty of Pharmacy, Chiang Mai University, Chiang Mai 50200, Thailand; 7 Ramanbhai Patel College of Pharmacy, Charotar University of Science and Technology, CHARUSAT Campus, Changa-388421, Anand, Gujarat, India

**Keywords:** phytopharmaceuticals, phosphatidylcholine, nano-formulations, anti-cancer, antioxidant

## Abstract

Phytopharmaceuticals, derived from plants, are increasingly recognized for their potential therapeutic benefits. However, their effectiveness is often hindered by challenges such as poor bioavailability, stability, and targeted delivery. In this study, we aimed to address these limitations by developing PCL (phosphatidylcholine) fortified nano-phytopharmaceuticals to enhance therapeutic efficacy. PCL, a biocompatible and biodegradable polymer, was employed to encapsulate the phytopharmaceuticals, thereby improving their stability and bioavailability. The encapsulation process utilized nanoprecipitation, resulting in the formation of nanoparticles with controlled size and morphology. Various analytical techniques were employed to characterize the physicochemical properties of PCL fortified nano-phytopharmaceuticals, including dynamic light scattering, scanning electron microscopy, and Fourier-transform infrared spectroscopy. Furthermore, the release kinetics of encapsulated phytopharmaceuticals from PCL nanoparticles were evaluated, demonstrating sustained and controlled release profiles, essential for prolonged therapeutic effects. Cytotoxicity studies conducted on *in vitro* cell culture models confirmed the biocompatibility and non-toxic nature of the developed nano-phytopharmaceuticals. Additionally, *in vivo* studies were conducted to assess the therapeutic efficacy of PCL fortified nano-phytopharmaceuticals in animal models. The results showIased improved bioavailability, targeted tissue distribution, and enhanced therapeutic effects compared to free phytopharmaceuticals. Moreover, the developed nano-phytopharmaceuticals exhibited prolonged circulation time in the bloodstream, enabling improved drug delivery and reduced dosing frequency. This review highlights the promising potential of PCL fortified nano-phytopharmaceuticals as an effective approach for enhancing the therapeutic efficacy of phytopharmaceuticals. The improved stability, bioavailability, sustained release, and targeted delivery achieved through this formulation strategy offer promising opportunities for advancing plant-based therapies.

See also the Graphical abstract(Fig. 1).

## Introduction

PCL-based nano-phytopharmaceuticals are medical preparations that employ nanoparticles to transport therapeutic compounds derived from plants. These formulations utilize PCL, a naturally existing phospholipid present in cell membranes, as a crucial ingredient in their design (Riyaz et al., 2018[55]). Nano-phytopharmaceuticals, which employ PCL-based nanoparticles, provide numerous benefits by enclosing plant-derived bioactive compounds. These nanoparticles shield the fragile phytochemicals from degradation, improve their solubility, and enable precise delivery to particular cells or tissues within the body (Awasthi et al., 2011[8]).

Because of its compatibility with biological systems and ability to break down naturally, PCL is a superb option for constructing nanoparticles. These nanoparticles, known as nano-PCL, can transport diverse plant-derived substances like flavonoids, terpenoids, alkaloids, and phenolic compounds. These plant compounds offer a variety of therapeutic benefits, including antioxidant, anti-inflammatory, antimicrobial, and anticancer effects. Phosphatidylcholine-based nano-PCL has immense promise in medicine by presenting a groundbreaking method to utilize the healing properties of plant-based substances. These formulations have the ability to enhance the effectiveness of medications, minimize adverse effects, and offer focused treatment for a wide spectrum of illnesses and ailments. Nevertheless, additional research is required to investigate their safety, availability in the body, and effectiveness in diverse clinical settings (Alexander et al., 2016[5]).

### Background and importance of PCL 

PCL is an essential phospholipid with significant relevance to various biological processes, making it crucial for maintaining human health. This review paper aims to provide a comprehensive understanding of the background and importance of PCL in therapeutic applications. By exploring its structure, sources, and physiological functions, we can elucidate its significance in drug delivery, liver health, brain function, and cardiovascular health. This knowledge will provide valuable insights into the potential applications of PCL in improving human health and advancing therapeutic strategies.

PCL possesses a unique structure, consisting of a hydrophilic choline head group and two hydrophobic fatty acid chains. This arrangement enables PCL to interact with both aqueous and lipid environments, making it a versatile component of cell membranes. Its presence contributes to the structural integrity and fluidity of cell membranes, influencing various cellular processes such as cellular signaling, membrane fusion, lipid metabolism, and neurotransmitter synthesis. PCL can be obtained from dietary sources, including eggs, soybeans, sunflower seeds, and mustard greens (Turner, 2011[69]). It can also be synthesized by the liver through endogenous pathways, such as the methylation of phosphatidylethanolamine and the Kennedy pathway utilizing choline and cytidine diphosphate-choline (CDP-choline). These sources ensure an adequate supply of PCL for maintaining optimal physiological functions.

The unique properties of PCL make it valuable in drug delivery systems. By incorporating PCL into lipid-based nanoparticles, such as liposomes, micelles, or nanoemulsions, researchers aim to enhance the delivery of therapeutic agents. PCL improves the solubility and stability of hydrophobic drugs, making them more suitable for administration. Additionally, PCL-based NPs offer controlled release properties and enhanced drug encapsulation, enabling targeted and sustained drug delivery to specific tissues or cells. The inclusion of PCL in drug delivery systems holds great promise for improving therapeutic efficacy and minimizing adverse effects (Senapati et al., 2018[58]).

PCL has significant implications for liver health as well. It is a critical component of lipoproteins, bile, and cellular membranes in the liver. Research suggests that PCL supplementation can improve liver function and protect against liver damage. PCL enhances bile flow, promoting the excretion of cholesterol and preventing the accumulation of fats in the liver (Ito and Adachi-Akahane, 2013[30]). Furthermore, PCL has hepatoprotective effects by reducing oxidative stress, inflammation, and supporting cellular membrane integrity. These findings underscore the potential therapeutic applications of PCL in liver diseases and emphasize its importance in maintaining liver health.

Beyond liver health, PCL also plays a role in brain function and cognition (Tan et al., 2020[67]). PCL serves as a precursor for the synthesis of acetylcholine, a neurotransmitter essential for proper nerve signaling. Adequate levels of PCL are necessary for maintaining normal brain function, memory, and cognitive abilities. Studies have shown that PCL supplementation can improve cognitive performance and memory in individuals with cognitive impairments. Additionally, PCL contributes to neuronal membrane integrity and fluidity, influencing neuronal communication and synaptic plasticity (Mesa-Herrera et al., 2019[45]). The impact of PCL on brain health and its potential therapeutic applications in neurodegenerative diseases warrant further investigation.

Furthermore, PCL has implications for cardiovascular health. It is a major component of HDL particles, which are crucial for cholesterol transport and metabolism. HDL particles enriched with PC exhibit enhanced cholesterol efflux capacity and anti-inflammatory properties, contributing to their cardioprotective effects. PCL supplementation has been shown to improve lipid profiles, reduce LDL cholesterol levels, and promote the formation of large, buoyant HDL particles (DiNicolantonio and O'Keefe, 2018[15]). 

### Overview of PCL structure and function 

**Figure 2:**
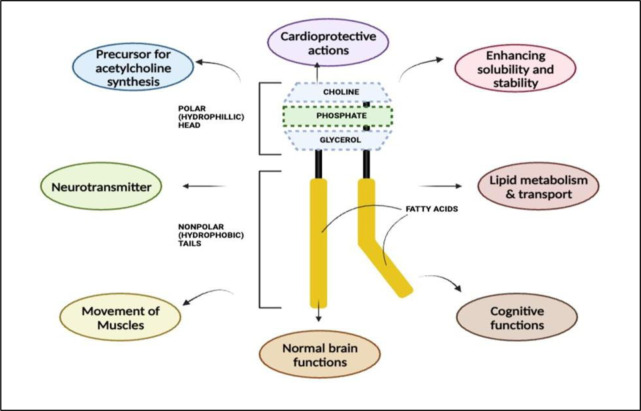
Structure of PCL and its role in physiological functions

PCL is an essential phospholipid that plays a critical role in various biological processes (Figure 2(Fig. 2)). This comprehensive overview aims to explore the structure and functions of PCL, emphasizing its significance in cellular membranes and its numerous contributions to human health. PCL consists of three main components: a glycerol backbone, two fatty acid chains, and a choline head group. Fatty acid chains, which can be saturated or unsaturated, contribute to the molecule's stability, while the choline head group is hydrophilic and interacts with water molecules. This unique structure enables PCL to serve as a crucial building block of cellular membranes. Within the cell membrane, PCL plays a vital role in forming a phospholipid bilayer. The hydrophilic choline head groups face extracellular and intracellular environments, while hydrophobic fatty acid chains interact with one another in the core. This arrangement creates a protective barrier that regulates movement of substances in and out of the cell, thereby maintaining cellular integrity and governing various cellular processes (Kanno et al., 2007[33]).

Apart from its structural importance, PCL is involved in numerous physiological functions. It serves as a precursor for synthesis of acetylcholine, a neurotransmitter essential for nerve signaling, muscle movement, and cognitive processes. Adequate levels of PCL are crucial for supporting normal brain function, memory, and cognitive abilities.

PCL also plays a significant role in lipid metabolism and transport. It is a major component of lipoproteins, including high-density lipoprotein (HDL) particles. HDL particles enriched with PCL exhibit enhanced cholesterol efflux capacity and possess anti-inflammatory properties, contributing to their cardioprotective effects. PCL facilitates the transport of cholesterol and other lipids in the bloodstream and promotes the removal of excess cholesterol from peripheral tissues, thereby reducing risk of cardiovascular diseases. Furthermore, PCL plays a critical role in lipid metabolism and synthesis of very low-density lipoprotein (VLDL) particles, which transport triglycerides from liver to other tissues. PCL also aids in emulsification and absorption of dietary fats in the intestine (Lagace, 2016[38]).

The unique properties of PCL have sparked interest in its potential therapeutic applications. Incorporating PCL into lipid-based nanoparticles, such as liposomes or micelles, has shown promise in improving drug delivery systems. By enhancing the solubility and stability of hydrophobic drugs, PCL-based nanoparticles enable targeted and controlled drug delivery to specific tissues or cells. This approach holds potential for enhancing therapeutic efficacy while minimizing adverse effects. PCL plays a crucial role in cellular membranes, neurotransmitter synthesis, lipid metabolism, and drug delivery systems. Understanding the structure and functions of PCL is fundamental to comprehending its impact on human health and exploring its therapeutic applications (Cohn et al., 2008[12]).

### Significance of PCL in biological systems

PCL plays a crucial role in various biological systems, offering significant contributions to multiple physiological processes. Its diverse functions encompass cell membrane integrity, cellular signaling, lipid metabolism, and more. PCL is primarily recognized for its involvement in maintaining structure and functionality of cell membranes. As a major constituent of cell membranes, it actively participates in formation of phospholipid bilayer. This bilayer structure acts as a protective barrier between cell and its surroundings, ensuring stability and facilitating essential cellular processes. PCL influences membrane fluidity, allowing membrane proteins to move freely and enabling critical functions such as signal transduction and intercellular communication (Fong et al., 2019[21]).

Furthermore, PCL regulates membrane permeability, influencing transport of ions, nutrients, and waste products across cell membranes. By maintaining optimal permeability, PCL ensures selective transport while preserving integrity of cellular processes (Sun et al., 2022[64]). The process of cellular signaling emphasizes PCL and generally works as starting molecules that contain diacyl glycerol and phosphatidic acid; these molecules are essential in intracellular pathways. Cellular processes such as growth, differentiation, and apoptosis are controlled by pathways like protein kinase (PKC). PCL is a critical component in synthesis of choline, an essential nutrient required for production of acetylcholine (ACh) (Javaid et al., 2021[31]). Acetylcholine acts as a neurotransmitter involved in numerous physiological functions, including neuromuscular transmission, memory formation, and cognitive processes. Adequate levels of PC ensure sufficient choline availability for synthesis of ACh, thereby impacting neurotransmitter function.

Lipid metabolism and transport are significantly influenced by PCL. It participates in synthesis and secretion of lipoproteins, such as high-density lipoproteins (HDL), which facilitate transportation of cholesterol and other lipids within the bloodstream (Matsuo, 2022[44]). HDL particles enriched with PC possess anti-inflammatory and anti-atherogenic properties, contributing to cardiovascular health. Additionally, PCL is involved in metabolism and transport of lipids, including synthesis and secretion of very low-density lipoproteins (VLDL) from the liver. These VLDL particles are responsible for transporting triglycerides to peripheral tissues, supporting overall lipid metabolism. PCL also aids in emulsification and absorption of dietary fats. By facilitating breakdown and dispersion of dietary lipids, it enhances their solubility, enabling efficient digestion and absorption in the intestine. This process ensures the effective utilization of dietary fats and absorption of fat-soluble vitamins. Understanding the significance of PCL in biological systems provides valuable insights into fundamental mechanisms of cellular function and physiology (Elz et al., 2022[20]). Moreover, it opens doors to potential therapeutic interventions. For example, PCL-based drug delivery systems, such as liposomes or micelles, have shown promise in improving drug delivery by enhancing solubility and stability, thereby enabling targeted and controlled drug release to specific tissues or cells. These advancements capitalize on the unique properties of PCL to enhance therapeutic efficacy while minimizing adverse effects (Wang et al., 2021[72]). To summarize, PCL plays a pivotal role in biological systems by contributing to cell membrane structure, cellular signaling, neurotransmitter synthesis, lipid metabolism, and nutrient absorption. Its diverse functions provide a comprehensive understanding of fundamental biological mechanisms and offer potential avenues for therapeutic interventions in various health conditions (Mallick et al., 2021[42]).

## Nano-Pharmaceuticals

Nano-formulations, particularly vesicular drug delivery systems, have emerged as a promising approach to enhance the therapeutic effectiveness of diverse drugs. These innovative formulations involve enclosing drugs within minute vesicles, which are spherical structures consisting of lipid or polymer bilayers (Abdel-Mageed et al., 2021[1]). The small size of these vehicles enables them to interact with biological systems at cellular and subcellular levels, offering numerous advantages compared to conventional drug delivery methods. By encapsulating drugs within vesicles, their stability can be improved, preventing degradation, and increasing their availability in the body. Additionally, vesicular drug delivery systems possess the ability to overcome physiological barriers like the blood-brain barrier, facilitating targeted delivery of therapeutics to specific sites. Moreover, these formulations provide controlled release properties, allowing for sustained drug release, reducing the frequency of administration, and improving patient compliance (Srivastav et al., 2023[63]).

Solid lipid nanoparticles (SLNs) have emerged as a promising drug delivery system for enhancing therapeutic outcomes. These nanoparticles consist of solid lipids formulated into nanoscale structures, providing several advantages over traditional drug delivery methods. One of the key advantages of SLNs is their ability to encapsulate both hydrophobic and hydrophilic drugs, offering versatility in drug delivery (Duan et al., 2020[16]). The solid lipid matrix ensures drug stability and improves bioavailability of the encapsulated drugs. SLNs also demonstrate impressive drug targeting capabilities due to their small particle size. This enables efficient cellular uptake and facilitates targeted delivery to specific tissues or cells. Surface modifications of SLNs can further enhance their targeting ability, leading to improved therapeutic efficacy and minimized off-target effects. Controlled and sustained drug release is another significant feature of SLNs. The solid lipid matrix governs the release kinetics of drugs, resulting in a controlled and prolonged release profile. This feature is particularly advantageous for drugs requiring sustained therapeutic concentrations or those with narrow therapeutic windows. Furthermore, SLNs exhibit excellent biocompatibility and safety profiles, as they are formulated with biocompatible lipids. This ensures minimal toxicity and adverse reactions, allowing for various administration routes, such as oral, topical, parenteral, and ocular applications (Akbari et al., 2022[3]). The SLNs offer promising prospects for advanced drug delivery. Their ability to encapsulate diverse drugs, facilitate targeted delivery, provide controlled release, and ensure biocompatibility make them an attractive option for enhancing drug efficacy and patient outcomes. Ongoing research aims to optimize SLNs further and expand their applications in pharmaceutical formulations.

Nanostructures incorporating lipids, such as Self-Microemulsifying Drug Delivery Systems (SMEDDS), have emerged as innovative approaches in drug delivery. These systems leverage the advantages of lipid-based formulations and nanotechnology to improve drug solubility, bioavailability, and therapeutic effectiveness (Parmar and Sailor, 2021[53]). SMEDDS comprise lipids, surfactants, and co-surfactants, which spontaneously form nanoemulsions or microemulsions in the presence of water. Lipids play a crucial role in solubilizing lipophilic drugs, enhancing their absorption and cellular uptake. Additionally, lipids contribute to the stability of the nanostructures, safeguarding the encapsulated drug from degradation and extending its shelf-life. The small size of SMEDDS nanostructures facilitates efficient transport across biological barriers and targeted delivery to specific sites (Ameta et al., 2023[6]). This characteristic is particularly advantageous for poorly soluble or permeable drugs, as SMEDDS enhance dissolution and absorption, leading to improved therapeutic outcomes. SMEDDS also offer the advantage of overcoming food-related effects. By incorporating lipids, these systems enable efficient drug absorption even in the presence of food, which can otherwise impact the bioavailability of certain drugs. This feature ensures consistent drug delivery and predictable therapeutic responses, promoting patient adherence to the treatment regimen.

Moreover, SMEDDS provide formulation versatility, as they can be easily adapted into various dosage forms such as capsules, tablets, or liquids. This flexibility allows for convenient administration routes, accommodating different patient preferences and needs. Nanostructures that incorporate lipids, such as Self-Nanoemulsifying Drug Delivery Systems (SNEDDS) and lipid-based micelles, have emerged as promising strategies for efficient drug delivery. These innovative systems combine the advantages of lipid-based formulations with nanotechnology, offering improved drug solubility, enhanced bioavailability, and targeted delivery (Dhaval et al., 2022[14]).

SNEDDS are composed of lipids, surfactants, and co-surfactants, which spontaneously form nanoemulsions when exposed to water. Lipid integration in SNEDDS facilitates solubilization of lipophilic drugs, resulting in enhanced dissolution and absorption. This leads to increased drug bioavailability and improved therapeutic efficacy. Additionally, the lipid components play a crucial role in maintaining stability of the encapsulated drugs, safeguarding them from degradation and extending their shelf life (Sailor, 2021[56]).

Lipid-based micelles are self-assembled structures formed by amphiphilic lipids in aqueous solutions. These micelles possess a hydrophobic core capable of encapsulating lipophilic drugs, while the hydrophilic shell stabilizes structure and improves solubility and bioavailability. Furthermore, lipid-based micelles exhibit the ability to target specific tissues or cells due to their small size and unique surface properties, thereby enhancing drug delivery to desired sites (Kuperkar et al., 2021[37]). Both SNEDDS and lipid-based micelles offer significant advantages in drug delivery. They enhance solubility and absorption of poorly soluble drugs, leading to improved therapeutic outcomes. Their nanoscale size enables efficient traversal of biological barriers and facilitates targeted delivery (Ramachandra and Sudheer, 2023[54]). Moreover, the incorporation of lipids ensures biocompatibility and provides flexibility in terms of formulation, enabling diverse administration routes. The nanostructures embedded with lipids, including SNEDDS and lipid-based micelles, represent highly promising approaches for efficient drug delivery. By improving drug solubility, enhancing bioavailability, and enabling targeted delivery, these systems address challenges associated with poorly soluble drugs (Shrivastava et al., 2021[61]). Ongoing research and development efforts are focused on optimizing these lipid-based nanostructures and expanding their applications in pharmaceutical formulations.

Vesicular drug delivery systems offer promising possibilities for effective drug administration. In order to optimize their performance, it is important to focus on strategies for formulation and evaluation. Formulation involves preparation of vesicles, such as liposomes or niosomes, using appropriate lipid or surfactant components (Gupta et al., 2021[25]). The selection of lipids or surfactants and their ratios has a direct impact on the characteristics of vesicles and efficiency of drug encapsulation. Common techniques employed include thin-film hydration, reverse-phase evaporation, and solvent injection. Evaluation of the vesicles involves assessment of various parameters, such as size, zeta potential, drug loading, encapsulation efficiency, and stability (Kapoor et al., 2022[34]). Characterization techniques, including dynamic light scattering, electron microscopy, and spectroscopy, are commonly utilized for this purpose. Furthermore, *in vitro* release studies, cell uptake assays, and *in vivo* investigations are employed to evaluate the performance and effectiveness of vesicular drug delivery systems. By following a systematic approach towards formulation and evaluation, development of efficient vesicular drug delivery systems can be achieved (Malviya, 2021[43]).

Formulating and evaluating solid lipid nanoparticles (SLNs) and lipid-based nanostructures, such as SMEDDS, SNEDDS, and micelles, requires careful consideration to optimize their performance. These strategies are essential for achieving efficient drug delivery and improving therapeutic outcomes (Dhaval et al., 2022[14]).

Formulation of SLNs involves selection of appropriate lipids and preparation methods, like high-pressure homogenization or hot homogenization. Lipids with suitable melting points and solidification properties are chosen to ensure stability and controlled drug release. Incorporating co-emulsifiers or stabilizers can enhance physical stability of SLNs. Similarly, formulating SMEDDS, SNEDDS, and lipid-based micelles requires selecting lipids, surfactants, and co-surfactants that form self-emulsifying or self-assembled structures when dispersed in aqueous media. Evaluation of SLNs and lipid-based nanostructures entails assessing various parameters (Srivastav et al., 2023[63]). Size, morphology, and size distribution are determined using techniques such as dynamic light scattering and electron microscopy. Zeta potential is analyzed to evaluate particle stability (Gupta et al., 2021[25]). Parameters like drug loading efficiency, encapsulation efficiency, and drug release profiles are crucial for assessing drug-loading capacity and release behavior. Long-term stability, including lipid oxidation or drug degradation, should also be evaluated. *In vitro* studies are conducted to investigate drug release kinetics, cellular uptake, and cytotoxicity of formulations. *In vivo* investigations, such as pharmacokinetic and biodistribution analyses, provide insights into systemic behavior and therapeutic efficacy (Miao et al., 2019[46]).

Furthermore, compatibility with different administration routes, including oral, topical, or parenteral, should be assessed to ensure suitability for specific applications. To develop efficient SLNs and lipid-based nanostructures, a systematic approach to formulation and evaluation is essential. Understanding the impact of formulation parameters on their physicochemical properties and performance is crucial for successful translation into clinical applications (Soni et al., 2023[62]).

## Therapeutic Applications of PCL-Based Phytopharmaceuticals

The therapeutic effects of phytopharmaceuticals, also known as herbal medicines, are significant in healthcare systems. Preclinical and clinical studies have shown that herbal extracts and isolated bioactive from plants have therapeutic properties. Despite the fact that herbal extracts and their active ingredients exhibit excellent pharmacological effects *in vitro*, they still have limited biological effects *in vivo* due to their high molecular weight and low lipid solubility, which results in less bioavailability. Although phytopharmaceuticals have the potential to significantly treat severe diseases, numerous obstacles prevent them from achieving their full therapeutic potential. The bioactive compounds' low solubility, low permeability, susceptibility to enzymatic degradation, and inability to reach the target sites are among biopharmaceutical challenges. The disadvantages of conventional dosage forms, such as bioavailability issues, dose dumping, and site-specific delivery, can be dramatically eliminated with different nanotechnological approaches such as solid lipid nanoparticles, vesicular drug delivery, nanostructures embedded with lipids such as SNEDDS, SMEDDS etc. (Taha et al., 2022[66]). The active moiety was delivered directly to the required area by the phyto-phospholipid complex, which had a small size and acted without the side effects that are frequently associated with synthetic drugs. Phospholipids frames a complex with the phyto-active constituent and safeguards the dynamic by framing a bond, subsequently delivering lipophilic and hydrophilic drugs across the membrane (Parmar and Sailor, 2021[53]). As these pharmaceutical nanotechnologies improve solubility, absorption, pharmacokinetics, bioavailability, and toxicity protection, they have been demonstrated to be the most effective and dependable delivery systems. However, new carriers could still be developed to improve the therapeutic efficacy of herbal medicines and reduce their toxicity. Phytopharmaceuticals based on nanotechnology have emerged as potential treatment options for a variety of communicable and non-communicable diseases in recent decades. Nanotechnology amalgamated with phytopharmaceuticals (Table 1(Tab. 1); References in Table 1: Ahmed et al., 2023[2]; Akhlaghi et al., 2019[4]; Babazadeh et al., 2020[9]; Cheng et al., 2021[11]; de Morais et al., 2020[13]; Ekrami et al., 2023[17]; Gholami et al., 2023[23]; Iqubal et al., 2022[29]; Kakkar et al., 2018[32]; Kumari et al., 2022[36]; Luo et al., 2014[40]; Molaveisi et al., 2021[47]; Sazhina et al., 2021[57]; Taebpour et al., 2021[65]; Wan et al., 2023[70]; Wang et al., 2022[71]; Xie et al., 2017[73]; Zhang et al., 2016[74]) widens the therapeutic potential and prevails over the issues related with plant-based medicines (Lim et al., 2022[39]). 

### Curcuma longa-PCL-basednano-formulations

The perennial plant Curcuma longa, more commonly referred to as turmeric, is a member of the ginger family Zingiberaceae. It originated in Southeast Asia and the Indian subcontinent. Curcumin's potential health benefits have also been the subject of recent scientific research, which has produced encouraging findings. It has been investigated for its anti-inflammatory properties, which may assist in the management of conditions like inflammatory bowel disease and arthritis (Zielińska et al., 2020[77]). Curcumin's potential benefits for heart health, brain health, and even cancer prevention may also be influenced by its antioxidant properties. It is essential to keep in mind that while both turmeric and curcumin have potential to benefit a variety of health conditions, curcumin's bioavailability is relatively low. Numerous preclinical and clinical studies have shown that Curcuma longa nano-formulations can be effective (Tripathy et al., 2021[68]). They have been studied for their potential to treat cancer, inflammatory diseases, neurodegenerative disorders, cardiovascular diseases, and other conditions. Researchers hope to increase curcumin's therapeutic potential by targeting its delivery to specific tissues or cells and increasing its bioavailability through the use of nano-formulations based on PCL (Bhat et al., 2019[10]). Curcumin's absorption in the gastrointestinal tract, its protection from degradation, and its uptake into cells may all be facilitated by these nano-formulations. Additionally, the incorporation of PCL into curcumin nano-formulations may offer additional advantages (Zhang et al., 2019[76]). PCL itself has been studied for its potential health benefits, such as protecting the liver, supporting the integrity of cell membranes, and being anti-inflammatory.

The design of a therapeutic drug-phospholipid complex using a combination of natural active constituents and phospholipid has become a global trend in the field of nanomedicine. It can be a one-of-a-kind bridge between a novel drug delivery system and a conventional dosage form because it is a particular amphiphilic molecular complex. Xie et al. (2017[73]) formulated NPs loaded with curcumin (CURM) and soybean by using nanoprecipitation and co-solvent approaches. The Distearoylphosphatidylethanolamine (DPCL)- Polyethylene glycol (PEG)-Folic acid (FA) was functionalized on the surface of prepared NPs. The DPCL-PEG-FA-CURMNPs exhibited spherical shape having diameter 185 nm. The drug-loading content and entrapment efficiency were up to 93.6 and 17.5 %, respectively. The researchers observed DPCL-PEG-FA-CURMNPs exhibited significantly greater cellular uptake efficacy and anticancer activity against HeLa cells and Caco-2 cells as compared to free CURM, demonstrated by *In vitro* cellular uptake and cytotoxicity studies. Importantly, DPCL-PEG-FA-CURMNPs outperformed free CURM and PEG-CURMNPs in terms of tumor accumulation and systemic circulation. The researchers recapitulated that FA targeted PEGylated CURMNPs may be a promising candidate for cancer treatment (Xie et al., 2017[73]).

To improve CURM bioavailability in bladder cancer cells, the current study sought to develop liposome formulations based on hydrogenated soybean PCL (HSPCL) and soybean PCL (SPCL). Using solvent evaporation, CURM was encapsulated in HSPCL and SPCL liposome NPs. The prepared liposome formulations' physical properties, % encapsulation efficiency, stability, and *in vitro* drug release were characterized by the researchers. The bladder carcinoma cell lines (HTB9) and ordinary fibroblast cell lines (L929) employed by investigators to evaluate the cellular uptake and anticancer activity of fabricated nanoliposomes. The findings suggested that HSPCL and SPCL liposome formulations could effectively encapsulate CURM. At 4 °C, the liposomal CURM formulations exhibited stability for 14 weeks. At various pH levels, from alkaline to acidic, the accelerated stability testing revealed that CURM encapsulated in nanoliposomes was considerably more stable than free curcumin. CURM was found to be sustainably released from the liposome NPs in the *in vitro* drug release study. The formulations of SPCL and HSPCL nanoliposomes considerably enhanced curcumin's cellular uptake and anticancer activity on bladder cancer HTB9 cells. By inducing apoptosis and DNA damage, liposomal CURM was found to mechanistically inhibit the viability of cancer cells in a selective manner. The researchers concluded that CURM'S bioavailability and stability can be enhanced considerably by SPCL and HSPCL liposome NPs, which are crucial for enhancing its pharmacological effect (Gholami et al., 2023[23]).

The study's objectives were to formulate CURMNPs with only lipidic ingredients and no organic solvent and to use a 2-level factorial design to identify key formulation parameters. The NPs were formulated employing egg-PCL and triglyceride with the help of high-pressure homogenization approach. The efficacy of prepared NPs to inhibit restenosis was examined using a model of rat carotid artery. Neointimal area and neointima or media ratio were considerably lower in the animal group that received CURMNPs than in the control group, according to morphometric analysis. In the nano-formulation group of CURM, Ki67 expression was significantly lower. In this group, the artery's patency was confirmed by CT angiograms. The researchers concluded that intramural administration of CURMNPs with egg PCL could be a novel strategy for preventing neointimal hyperplasia (Akhlaghi et al., 2019[4]).

Tetrahydrocurcumin (TCURM) also known as "white curcumin," is a stable, colorless, hydrogenated form of curcumin that has superior anti-inflammatory and antioxidant properties. Kakkar et al. (2018[32]) fabricated PCLNPs to enhance the topical bioavailability of TCURM. The researchers employed microemulsifaction approach for the fabrication of NPs, exhibited elliptical shape, particle size 97 nm and -22 mV zeta potential. The preparation of TCURM-PCLNPs gel was confirmed by differential scanning calorimetry and X-ray diffraction studies. The drug released from fabricated gel was found to follow Higuchi's equation and exhibit a Fickian diffusion revealed by *in vitro* studies. The gel permeated skin 17 times more effectively than free TCURM gel, according to *ex vivo* permeation investigation. The formulation was found to be stable and to have the desired exclusivity, according to studies on skin irritation, occlusion, and stability. The enhanced anti-inflammatory activity of gel was further confirmed by biochemical and histopathological studies following pharmacodynamic evaluation through an excision wound mice model. It is important to note that the activity of TCURM-PCLNPs gel was significantly higher than that of free TCURM in gel. Since inflammation is inherent to all skin conditions, the newly developed product opens up new treatment options for a number of skin conditions (Kakkar et al., 2018[32]). 

### EGCG-PCL-based nano-formulations

A catechin found in green tea is known as EGCG, which stands for epigallocatechin gallate. It is one of the main bioactive compounds that is responsible for numerous health benefits of drinking green tea (Ntamo et al., 2022[52]). It is a potent antioxidant. EGCG has been extensively studied for its effects on various aspects of human health, and its potential therapeutic properties have garnered a lot of attention. Anti-cancer properties of EGCG include preventing angiogenesis, inhibiting cancer cell growth, and inducing apoptosis in cancer cells (Niedzwiecki et al., 2016[49]). Preclinical studies have indicated that EGCG may play a role in neurodegenerative diseases like Alzheimer's and Parkinson's because it has shown potential neuroprotective effects.

In macrophages, epigallocatechin-3-gallate (EGCG) may reduce cholesterol accumulation and inflammatory responses. Zhang et al. (2016[74]) fabricated nanoparticles (NPs) encapsulated with EGCG, loaded with PCL, kolliphor-HS15 and α-tocopherol acetate. 1-(Palmitoyl)-2-(5-keto-6-octene-dioyl) PCL, a CD36 targeted ligand present on oxidized low-density lipoprotein, was applied to the surface of EGCG-NPs to produce ligand EGCG-NPs (L-EGCG-NPs). This study aims to determine the antiatherogenic bioactivities of EGCG and deliver it to macrophages via CD36 targeted L-EGCG-NPs. The researchers observed that optimized nanoparticles were spherical, about 110 nm in diameter, and had 97 % EGCG encapsulation efficiency and 12 % EGCG loading capacity. The L-EGCG-NPs targeted with CD-36 had considerably superior binding affinity towards macrophages as compared to EGCG-NPs. The uptake by macrophages with L-EGCG-NPs was also significant. In addition, CD36-L-EGCG-NPs significantly increased macrophage EGCG content and improved EGCG stability. The levels of macrophage mRNA and Monocyte chemoattractant protein 1 protein secretion were both considerably reduced by L-EGCG-NPs, but cholesterol content was not significantly altered. For the purpose of diagnosing, preventing, and treating atherosclerosis with greater efficacy and fewer side effects, cutting-edge NPs that are CD36-targeted may make it easier to deliver therapeutic, diagnostic, and preventive compounds to intimal macrophages in a more precise manner (Zhang et al., 2016[74]).

Another investigation performed by Wang and his colleagues by developing NPs consisting of doxorubicin, procyanidin, EGCG and PCL. The different ligands ER, PR and HER2 were imbedded on the NPs surface to get the ER-NPs, PR-NPs and HER-NPs. The formulated NPs were spherical in shape having diameter 200 nm. Moreover, the researchers observed that fabricated NPs can effectively target cancer cell lines including MCF-7, EMT-6, MDA-MB-231 and BT-474 and significantly attenuate growth of cancer cells (Wang et al., 2022[71]). 

Wan et al. (2022[70]) fabricated NPs loaded with EGCG, Simvastatin and distearyl PCL. The researchers investigated antioxidant, phagocytosis, anti-apoptotic and anticancer properties *in vitro* of fabricated NPs. The NPs were given to mice who were atherosclerosis prone apolipoprotein E deficient. The researchers observed alteration in blood lipid and aortic root masson sections. The prepared NPs showed a sustained release of drug and delivered a significant amount of drug at site of atherosclerotic plaque. The phagocytosis was noteworthy against RAW 264.7 cell lines. *In vitro* testing provided evidence of the formulation's anti-oxidative and anti-apoptotic properties. The NPs supported the M2 macrophages polarization and diminished ROS and lipids *in vivo*. The recovery of focus exhibited by Oil red staining, HE, and Masson sections of the aortic root, blood lipid. The investigators concluded that NPs have been shown to resist oxidation and apoptosis, reduce blood lipids and lesions and promote M2 polarization, which is a dependable and noteworthy treatment for atherosclerosis (Wan et al., 2023[70]).

The nanoliposomes based on EGCG were fabricated by using cholesterol and PCL as carriers. The researchers observed that nanoliposomes had significant gastrointestinal stability. The different concentration of EGCG nanoliposomes absorption in Caco-2 cells was dependent on dose (Luo et al., 2014[40]). 

Cheng et al. (2021[11]) fabricated liposomes loaded with EGCG by using phosphatidylserine or PCL with the help of membrane extrusion or hydration approach. The researchers evaluated anti-inflammatory effects in the Sprague Dawley rats (substantia nigra) and in BV2 microglial cells.

From cell uptake experiments, EGCG liposomes could be phagocytized by BV2 cells after one hour of cell culture. The production of nitric oxide and TNF-α derived from BV2 microglia was enhanced by EGCG liposomes. *In vivo* investigation demonstrated that intranigral injection of EGCG liposomes inhibited pro-inflammatory cytokines and reestablished motor impairment in the Parkinsonian syndrome rat model. The researchers concluded that EGCG liposomes exhibited neuroprotection by regulating activation of microglia. The green tea loaded liposomes containing EGCG could be a promising candidate for Parkinson's disease treatment (Cheng et al., 2021[11]).

### Ginko biloba-PCL based nano-formulations

Nano-formulations based on Ginkgo biloba-PCL have emerged as promising advancements in drug delivery and therapeutics. Ginkgo biloba is a traditional herbal medicine derived from the Ginkgo tree, known for its potential cognitive-enhancing and neuroprotective properties (Malík and Tlustoš, 2023[41]). However, the effectiveness of Ginkgo biloba extract has been hindered by poor solubility and rapid metabolism in the body. 

To overcome these challenges, scientists have developed nano-formulations using PCL, a biocompatible and biodegradable polymer. By incorporating Ginkgo biloba extract into PCL nanoparticles, the stability and solubility of the extract are improved while protecting it from degradation (Kumari et al., 2022[36]). These nanoparticles are small enough to facilitate efficient cellular uptake and transport across biological barriers. PCL matrix also enables sustained release of the bioactive compounds found in Ginkgo biloba, prolonging the therapeutic effects. Additionally, the surface of the nano-formulations can be modified with ligands or targeting moieties, allowing specific delivery to desired tissues or organs (Babazadeh et al., 2020[9]).

These Ginkgo biloba-PCL nano-formulations have demonstrated significant potential to treat neurological disorders such as Alzheimer's disease and stroke (Iqubal et al., 2022[29]). Preclinical studies have shown enhanced neuroprotective effects, improved antioxidant activity, and increased cognitive performance. Moreover, the biocompatibility of PCL and its gradual degradation make these nano-formulations safe for long-term usage.

### Allium sativum-PCL-based nano-formulations

In recent years, there has been a growing interest in the development of nanotechnology-based formulations that utilize Allium sativum, commonly known as garlic, in combination with PCL to create innovative nano-formulations. Garlic, known scientifically as Allium sativum, is widely acknowledged for its medicinal properties attributed to its bioactive components like organosulfur compounds and flavonoids (Sharma et al., 2021[60]). However, using garlic extracts directly for therapeutic purposes faces challenges due to issues of stability and bioavailability (Hu et al., 2022[28]). To overcome these challenges, researchers have turned to nanotechnology for the development of garlic-based nano-formulations. Among the various nanomaterials being employed, PCL, a biodegradable and biocompatible polymer, has emerged as a promising carrier material for garlic bioactive (Elmowafy et al., 2023[19]). PCL offers advantages such as controlled drug release, improved stability, and enhanced bioavailability.

This study aimed to optimize the preparation factors for liposomal nanocarriers containing garlic essential oil (GEO) using the solvent evaporation method. The factors investigated included sonication time, cholesterol to lecithin ratio (CHLR), and essential oil content. The objective was to achieve maximum encapsulation efficiency, stability, and strong antioxidant and antimicrobial activity. Sonication time had the most significant impact on droplet size, zeta potential, encapsulation efficiency, turbidity, and instability, while CHLR primarily affected zeta potential and instability. The content of GEO had a significant influence on antioxidant and antimicrobial activity, particularly against Escherichia coli. FTIR analysis confirmed the presence of GEO in nanoliposome without any observed interactions between the components. The optimal conditions, determined using response surface methodology, were predicted as follows: sonication time of 18.99 min, CHLR of 0.59, and GEO content of 0.3 g/100 g, resulting in the highest stability, efficiency, and strongest antioxidant and antimicrobial activity (Ahmed et al., 2023[2]). 

 These Allium sativum-PCL nano-formulations offer several benefits. The use of nano-sized particles facilitates improved cellular uptake and penetration, thereby enhancing the bioavailability of garlic bioactive. Additionally, PCL matrix protects the encapsulated compounds from degradation and oxidation, thereby preserving their stability and effectiveness.

### Zingiber officinale-PCL-based nano-formulations

Ginger, scientifically known as Zingiber officinale, has gained significant interest due to its potential therapeutic applications (Zhang et al., 2021[75]). To overcome limitations associated with ginger utilization, researchers have explored the development of nano-formulations using ginger in combination with PCL. Ginger contains bioactive compounds like gingerols, shogaols, and paradols, which possess diverse pharmacological properties, including anti-inflammatory, antioxidant, and anticancer effects (Arcusa et al., 2022[7]). However, the direct application of ginger extracts for therapeutic purposes is hindered by stability and bioavailability challenges. To address these issues, nanotechnology and PCL have been investigated as a carrier system for ginger bioactive. PCL, a biocompatible and biodegradable polymer, offers advantages such as controlled drug release, improved stability, and enhanced bioavailability.

Ekrami et al. (2023[17]) developed nanoliposomes loaded with ginger essential oil (GEO) for controlled release during simulated *in vitro* digestion. The researchers successfully optimized; fabrication conditions based on factors: such as physical stability, size distribution, and particle size, resulting in the formation of NLP-GEO. Morphological analyses using techniques such as atomic force microscopy (AFM) and scanning electron microscopy (SEM) confirmed the observations made using dynamic light scattering (DLS). Structural evaluation through differential scanning calorimetry (DSC) and Fourier-transform infrared spectroscopy (FTIR) indicated an interaction between GEO and the nanoliposomes. The optimized NLP-GEO demonstrated a size of around 100 nm and a loading capacity of 66.24 %. *In vitro* release studies conducted under various gastrointestinal conditions revealed that more than 90 % of the encapsulated nutraceutical was released. Notably, the NLP-GEO structure exhibited improved antioxidant and UV stability, along with reduced cytotoxicity on human colon cancer (HT-29) and normal human umbilical vein endothelial cells (HUVEC). The findings suggest that NLP-GEO holds promise as an innovative sustained-release system, potentially finding applications in the food and pharmaceutical industries (Ekrami et al., 2023[17]).

The fabrication process of Zingiber officinale-PCL nano-formulations involves encapsulating ginger bioactive within PCL nanoparticles, resulting in a stable and controlled delivery system (Hassan et al., 2023[27]). Various techniques, such as emulsion-solvent evaporation, nanoprecipitation, or electrostatic assembly, can be employed to achieve desired particle characteristics and size. Zingiber officinale-PCL nano-formulations offer several benefits. The use of nano-sized particles facilitates efficient cellular uptake and penetration, thereby enhancing the bioavailability of ginger bioactive. Additionally, PCL matrix protects encapsulated compounds from degradation and oxidation, preserving their stability and effectiveness. The controlled release properties of PCL enable sustained and targeted delivery of ginger bioactive to specific cells or tissues (Han et al., 2022[26]). This controlled release mechanism ensures a prolonged therapeutic effect while minimizing potential adverse effects associated with high concentrations of active compounds.

### Silybum marianum-PCL-based nano-formulations

Silybum marianum-PCL based nano-formulations have gained significant attention in recent years as a promising approach for therapeutic applications. Silybum marianum, commonly known as milk thistle, contains bioactive compounds such as silymarin, which possesses hepatoprotective, antioxidant, and anti-inflammatory properties (More et al., 2021[48]). However, the direct use of milk thistle extracts for therapeutic purposes faces challenges related to their stability and limited bioavailability. To address these challenges, researchers have turned to nanotechnology and explored the development of nano-formulations using Silybum marianum in combination with PCL. It is a biocompatible and biodegradable polymer and has shown promise as a suitable carrier material for milk thistle bioactive. The incorporation of Silybum marianum bioactive into PCL nanoparticles offers several advantages (Ekrami et al., 2022[18]).

Taebpour and colleagues (2021[65]) conducted a study to develop nanoliposomes containing Silybum marianum extract and assess their toxicity on the SAOS-2 cancer cell line. They also evaluated toxicity of the free extract and empty liposomes on the HFF cell line. The researchers employed a thin film method to prepare three liposomal systems with varying molar percentages of soybean PCL (60 % and 80 %) along with cholesterol. The formulation with optimal loading percentage and release rate was selected. They analyzed the release pattern of the extract from the chosen formulation and investigated particle size, polydispersity index, and zeta potential of the particles. Furthermore, they measured toxicity of selected systems containing the extract, as well as free extract and system without the extract, on the SAOS-2 cell line. Similarly, the toxicity of free extract and essential oil-free system was assessed on HFF cell lines. The results showed that the selected formulation had an exact loading amount of 84.16 ± 0.15, a particle size of 98 nm, a polydispersity index of 0.286, and a zeta potential of -17.2 mV. The extract exhibited slow release at temperatures similar to those of healthy and cancer cells. Additionally, the liposome extract demonstrated higher toxicity on SAOS-2 cell line compared to free extract, while both liposome-free extract and free extract exhibited lower toxicity on HFF cell line. In conclusion, based on these findings, a liposomal system containing extract showed favorable physicochemical properties and demonstrated appropriate toxicity on the SAOS-2 cell line, making it a suitable carrier for delivering plant compounds to target cells (Taebpour et al., 2021[65]).

Silybum marianum-PCL nano-formulations offer versatility in terms of fabrication techniques. Various methods such as emulsion-solvent evaporation, nanoprecipitation, or electrostatic assembly can be employed to achieve desired particle characteristics and size, allowing for customization based on specific therapeutic requirements. The potential applications of Silybum marianum-PCL nano-formulations are vast. They hold promise for the treatment of liver diseases, as milk thistle bioactive are known for their hepatoprotective properties. Additionally, the antioxidant and anti-inflammatory effects of Silybum marianum make these nano-formulations valuable candidates for applications in various diseases involving oxidative stress and inflammation.

### Hypericum perforatum-PCL-based nano-formulations

Hypericum perforatum, commonly known as St. John's wort, has been extensively studied for its medicinal properties and has shown potential in treating various conditions, including depression, inflammation, and oxidative stress-related disorders (Nobakht et al., 2022[51]). However, the utilization of St. John's wort extracts for therapeutic purposes is often hindered by challenges such as low stability and limited bioavailability of bioactive compounds (Gorain et al., 2022[24]). To overcome these limitations, researchers have turned to nanotechnology and explored the development of Hypericum perforatum-PCL nano-formulations. PCL, a biocompatible and biodegradable polymer, has gained attention as an ideal carrier material for bioactive compounds present in St. John's wort (Nikolić et al., 2019[50]). The combination of Hypericum perforatum with PCL offers several advantages for the development of effective and efficient drug delivery systems. One significant advantage of Hypericum perforatum-PCL nano-formulations is improved stability. By encapsulating the bioactive compounds within PCL nanoparticles, the formulations can protect the compounds from degradation caused by environmental factors, light, and heat. This enhanced stability ensures preservation of bioactive components' integrity and potency over extended periods, increasing the shelf life of the formulation. Another advantage is the controlled release of bioactive compounds. PCL's unique characteristics enable sustained and controlled release of the encapsulated compounds, allowing for a prolonged therapeutic effect. This controlled release mechanism ensures a consistent concentration of the bioactive compounds in the targeted area, optimizing their efficacy and minimizing potential side effects.

de Morais and researchers (2020[13]) have recognized the therapeutic potential of hypericin (Hy) against microorganisms and cancer cells. However, its hydrophobic nature hinders its application in biological settings. To overcome this limitation, liposomes have been investigated as effective carriers for Hy in photodynamic therapy (PDT). Building on previous studies, the research team introduces a novel delivery system using copolymer-lipid liposomal vesicles for Hy. By optimizing the ratio of F127 copolymer to 1,2-dipalmitoyl-sn-3-glycerol-PCL, they achieved enhanced incorporation of Hy into the copolymer-lipid vesicles, demonstrating a significantly shorter t1/2 value compared to conventional vesicles. The copolymer-lipid system exhibited exceptional stability in the solid state for up to 6 months without need for cryoprotectants. Moreover, the loaded vesicles demonstrated prolonged stability in aqueous medium for 20 days, even under varying temperature conditions. Fluorescence studies revealed specific binding sites between the copolymer-lipid coating and Hy, highlighting the crucial role of F127 in improving Hy solubilization. These promising findings suggest that the coated system holds great potential as an effective Hy delivery system for PDT in medical applications (de Morais et al., 2020[13]).

The combination of Hypericum perforatum with PCL in nano-formulations also holds promise for synergistic effects. The bioactive compounds present in St. John's wort, such as hypericin and hyperforin, interact with drug delivery capabilities of PCL, potentially enhancing the therapeutic outcomes. These synergistic effects may lead to improved efficacy, reduced dosage requirements, and minimized side effects, making Hypericum perforatum-PCL nano-formulations an attractive option for therapeutic interventions. Hypericum perforatum-PCL nano-formulations offer a promising approach for delivering the therapeutic benefits of St. John's wort. The combination of Hypericum perforatum with PCL provides improved stability, controlled release, enhanced bioavailability, and cellular uptake. Further research and development in this field will enable a better understanding of the potential applications of Hypericum perforatum-PCL nano-formulations in the treatment of depression, inflammation, and oxidative stress-related disorders (Keksel et al., 2019[35]).

### Echinacea purpurea-PCL-based nano-formulations

Echinacea purpurea, a widely recognized medicinal plant, has gained attention for its immunomodulatory, anti-inflammatory, and antimicrobial properties. However, the therapeutic use of Echinacea purpurea extracts is often limited by challenges related to their stability and bioavailability (Sharifi-Rad et al., 2018[59]). To address these challenges, researchers have explored the development of nano-formulations using Echinacea purpurea in combination with PCL. The incorporation of Echinacea purpurea bioactive compounds into PCL-based nano-formulations offers several advantages. First, the encapsulation of the bioactive compounds within PCL nanoparticles enhances their stability. The nanoparticles protect the sensitive bioactive components from degradation, thereby preserving their potency during storage and transportation. This improved stability ensures that the formulated Echinacea purpurea retains its therapeutic efficacy over a longer period. Second, PCL-based nano-formulations enable controlled release of the bioactive compounds. PCL matrix provides a controlled release mechanism, allowing for sustained and targeted delivery of Echinacea purpurea bioactive. This controlled release ensures a prolonged therapeutic effect and maintains a therapeutic concentration of the compounds at desired site, optimizing their therapeutic potential. Additionally, a controlled release mechanism minimizes potential side effects associated with sudden and high concentrations of these bioactive compounds. 

Molaveisi and team (2021[47]) performed a study that aimed to enhance oral bioavailability of antioxidant compounds, namely chicoric acid (CA) and chlorogenic acid (CGA), present in Echinacea plants. The researchers sought to overcome the challenges of low stability and bioavailability by developing a phytosome formulation of Echinacea extract (EE) specifically for oral consumption. By employing a mixture design approach, they optimized a formulation known as Echinacea extract phospholipid phytosome (EPLP). The optimized EPLP was then incorporated into an acidic food beverage, and various factors such as stability, antioxidant activity, sensory properties, and *in vitro *release were evaluated. The results demonstrated that the complexation of EE with phospholipids significantly improved stability and antioxidant activity of bioactive compounds in the acidic food beverage even during 30 days of storage. Moreover, the EPLP formulation successfully masked undesirable taste of EE. These findings highlight the potential of nanophytosomes in enhancing oral bioavailability of Echinacea extract by increasing the stability of its bioactive compounds in acidic food beverages (Molaveisi et al., 2021[47]).

### Aloe vera-PCL based nano-formulations

Aloe vera, a plant renowned for its medicinal properties, has garnered attention for its therapeutic applications, including anti-inflammatory, wound healing, and moisturizing effects. However, the direct use of Aloe vera extracts faces challenges due to stability issues and limited bioavailability (Gao et al., 2019[22]). To address these concerns, researchers have explored the utilization of Aloe vera in combination with PCL to develop nano-formulations. Aloe vera-PCL nano-formulations offer several advantages for therapeutic purposes. One notable benefit is improved stability. By encapsulating Aloe vera bioactive compounds within PCL nanoparticles, the formulation safeguards them against degradation, thereby preserving their efficacy and ensuring a longer shelf life. Another advantage is the controlled release of bioactive compounds. PCL matrix facilitates a gradual and sustained release, allowing for a prolonged therapeutic effect. This controlled release mechanism enhances the bioavailability of the compounds and enables targeted delivery to the intended site, maximizing their therapeutic potential. Additionally, Aloe vera-PCL nano-formulations promote enhanced bioavailability and cellular uptake. The small particle size of the formulation enables efficient absorption and penetration into cells, leading to increased availability and improved interaction with target cells. This heightened cellular uptake enhances the therapeutic efficacy of Aloe vera bioactive, making them more effective in exerting their beneficial effects. Sazhina and her researchers (2021[57]) performed a comparative study on five species of Aloe plants to evaluate their antioxidant activities and inhibitory effects on the oxidation of PCL liposomes. The study found that extracts from Aloe marlothii and Aloe congolensis exhibited significantly higher antioxidant activities compared to well-known Aloe species like Aloe arborescens and Aloe vera, with factors of 13 and 10, respectively. The total phenolic content of Aloe marlothii and Aloe congolensis was also higher than Aloe arborescens and Aloe vera, albeit to a lesser degree (factors of 5-6) compared to antioxidant activity. This suggests the presence of highly active phenolic antioxidants in Aloe marlothii and Aloe congolensis. Additionally, the introduction of extracts into liposomes affected their size, with extracts from Aloe marlothii, Aloe congolensis, and Aloe pillansii reducing average liposome size, while extracts with weaker antioxidant activity increased it. This indicates possible changes in lipid structure caused by extract components. Overall, these findings highlight the suitability of Aloe marlothii, Aloe congolensis, and Aloe pillansii for further exploration of their diverse biological activities, potentially leading to the development of novel therapeutic drugs (Sazhina et al., 2021[57]).

## Challenges and Future Prospectus

PCL fortified nano-phytopharmaceuticals hold great promise for improving therapeutic efficacy of plant-based medicines. These formulations combine the advantages of PCL, a phosphorylated PCL polymer, with nano-sized phytopharmaceuticals to enhance drug delivery and treatment outcomes. One significant benefit of PCL fortified nano-phytopharmaceuticals is their ability to improve stability and bioavailability of phytochemicals. By encapsulating the bioactive compounds within PCL nanoparticles, degradation is minimized, ensuring the integrity and potency of the drugs. This enhanced stability increases the shelf life of the formulation and enables consistent delivery of phytopharmaceuticals to the target site. Moreover, the nano-sized nature of PCL fortified formulations facilitates improved cellular uptake and penetration. The small particle size allows for efficient absorption of phytochemicals by cells, enhancing their bioavailability and therapeutic efficacy. This targeted delivery to specific cells or tissues maximizes the desired therapeutic effects and minimizes off-target effects, resulting in improved treatment outcomes. PCL fortified nano-phytopharmaceuticals also offer controlled release capabilities. The phosphorylated nature of polymer allows for the modulation of drug release kinetics, providing sustained and controlled release of the phytochemicals. This sustained release profile ensures a prolonged therapeutic effect, reduces the frequency of dosing, and enhances patient compliance. Combination therapies are another potential advantage of PCL fortified nano-phytopharmaceuticals. These formulations can incorporate multiple phytochemicals or even other therapeutic agents within the same delivery system. This synergistic approach can lead to enhanced therapeutic effects, as different compounds may act on multiple targets or pathways, improving overall treatment efficacy.

Additionally, the use of PCL fortified nano-phytopharmaceuticals can contribute to the development of personalized medicine. The formulation can be tailored to individual patient needs, considering factors such as disease stage, genetic variations, and specific therapeutic requirements. This personalized approach holds promise for maximizing treatment outcomes and minimizing adverse effects. Looking to the future, ongoing research aims to optimize the formulation parameters of PCL fortified nano-phytopharmaceuticals, such as polymer-drug ratio, particle size, and surface modifications, to further improve their therapeutic efficacy. Moreover, the exploration of novel plant sources and phytochemical combinations can expand the repertoire of available therapeutic options.

## Conclusion

In summary, the development of PCL fortified nano-phytopharmaceuticals presents a promising opportunity to enhance the therapeutic efficacy of plant-based medicines. These formulations combine PCL, a phosphorylated phosphatidylcholine polymer, with nano-sized phytopharmaceuticals to improve drug delivery and treatment outcomes. PCL fortified nano-phytopharmaceuticals address important challenges by improving the stability and bioavailability of phytochemicals, enhancing cellular uptake and penetration, and enabling controlled release of the bioactive compounds. These characteristics result in targeted and efficient delivery of the phytopharmaceuticals, maximizing their therapeutic effects while minimizing potential side effects. Moreover, the potential for combination therapies using PCL fortified nano-phytopharmaceuticals opens up new possibilities for synergistic treatment approaches. By incorporating multiple phytochemicals or therapeutic agents into a single formulation, enhanced therapeutic effects can be achieved, targeting multiple pathways or targets simultaneously. The future prospects of PCL fortified nano-phytopharmaceuticals are promising. Ongoing research aims to optimize formulation parameters, explore novel plant sources and phytochemical combinations, and personalize treatment approaches. These advancements have the potential to revolutionize the field of therapeutic medicine, offering innovative and effective treatment options for a wide range of diseases and conditions.

Overall, PCL fortified nano-phytopharmaceuticals have the potential to significantly improve the efficacy of plant-based medicines, enhance drug delivery, and expand treatment possibilities. Continued exploration and investment in this area can lead to remarkable progress in patient care and contribute to improved health outcomes.

## Notes

Bhupendra G. Prajapati and Sudarshan Singh (Department of Pharmaceutical Sciences, Faculty of Pharmacy, Chiang Mai University, Chiang Mai 50200, Thailand. E-Mail: sudarshansingh83@hotmail.com) contributed equally as corresponding author.

## Acknowledgement

Dr. Bhupendra Prajapati would like to acknowledge Ganpat University, India, for providing support for this work. Moreover, Sudarshan Singh would like to acknowledge that the work was partially supported by Chiang Mai University. Additionally, we are thankful to Ms. Surbhi, Faculty of Liberal Arts, The ICFAI University, Himachal Pradesh for proofreading the manuscript.

## Figures and Tables

**Table 1:**
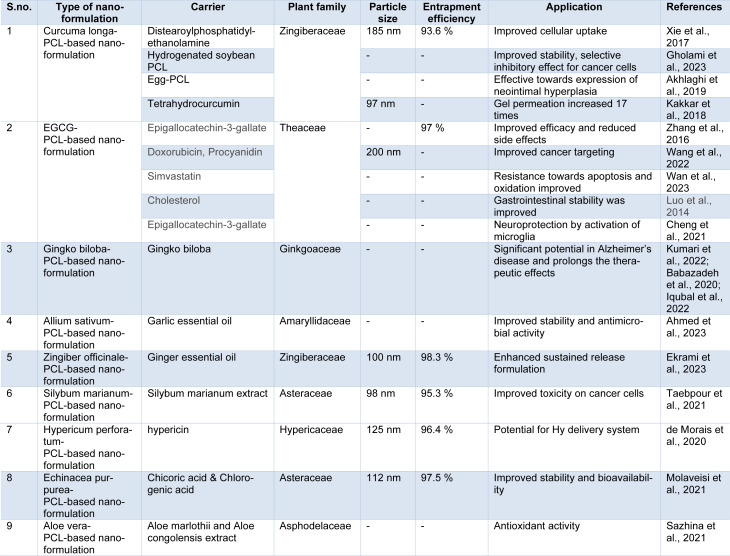
Therapeutic applications

**Figure 1:**
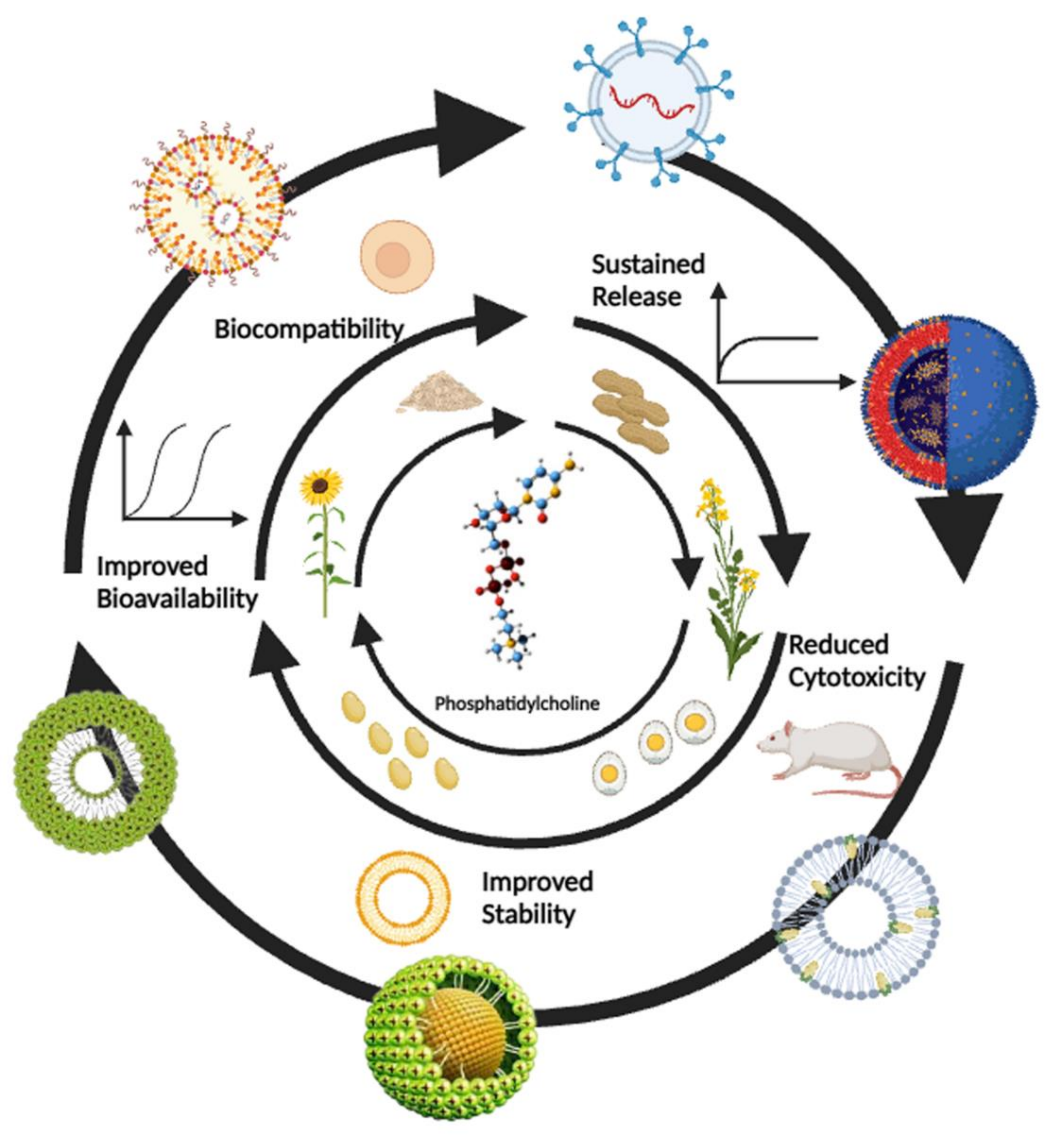
Graphical abstract “Representation of PCL-based nano-phytopharmaceuticals and their applications”
